# Engineering Graphene Oxide/Water Interface from First Principles to Experiments for Electrostatic Protective Composites

**DOI:** 10.3390/polym12071596

**Published:** 2020-07-18

**Authors:** Luca Valentini, Silvia Bittolo Bon, Giacomo Giorgi

**Affiliations:** 1 Dipartimento di Ingegneria Civile e Ambientale, Università degli Studi di Perugia, Strada di Pentima 4, 05100 Terni, Italy; silvia.bittolo@gmail.com; 2 Dipartimento di Ingegneria Civile e Ambientale, Università degli Studi di Perugia, Via G. Duranti 93, 06125, Perugia, & CNR-SCITEC, I-06123 Perugia, Italy

**Keywords:** graphene oxide, composite fibers, electrical properties, water interface, mechanical properties

## Abstract

From the global spread of COVID-19 we learned that SARS-CoV-2 virus can be transmitted via respiratory liquid droplets. In this study, we performed first-principles calculations suggesting that water molecules once in contact with the graphene oxide (GO) layer interact with its functional groups, therefore, developing an electric field induced by the heterostructure formation. Experiments on GO polymer composite film supports the theoretical findings, showing that the interaction with water aerosol generates a voltage output signal of up to −2 V. We then developed an electrostatic composite fiber by the coagulation method mixing GO with poly(methyl methacrylate) (PMMA). These findings could be used to design protective fabrics with antiviral activity against negatively charged spike proteins of airborne viruses.

## 1. Introduction

Electricity generation from the water–nanocarbon interface has been extensively investigated in the last years for blue energy harvesting purposes [1,2,3,4]. Between nanostructured carbons, graphene oxide (GO) is a versatile material due to its surface chemistry, which allows for its dispersion in water and the design and engineering of polymer composites and membranes [5,6,7,8]. From a first-principles point of view, very recently, it was demonstrated that GO in liquid water acquires a negative charge [2,9]. The combination of GO sharp edged structure and surface charge when dispersed into nonionic polymers was found to be efficient in antiviral activity [10]; thus, aerosols consisting of water droplets with a dimension in the range from 100 nm to 1 μm that cannot be stopped mechanically by the pores of filter fibers could be removed, for example, by the electrostatic interaction with GO based polymer fibers, making the face masks more efficient [11].

In this regard, the surface chemistry of GO allows its incorporation into polymer matrices such as a polycarbonate (PC) matrix [12], and nylon 6 [13] to increase the degradation and melting temperatures. Moreover, such nanocomposites can be further processed to obtain fabrics with added value in terms of mechanical strength and electrical conductivity.

In previous studies, for example, we investigated the mechanical excitation through falling water droplets and acoustic waves on reduced GO composites to convert the mechanical energy of the impact to electrical power [14,15], but the charging of the GO surface in contact with water when it is embedded into a polymer matrix, to our best knowledge, has not been investigated yet.

The combined provision of what is previously reported [10,11] requires calculation methods dedicated to optimize the surface charge of 2D materials and deposition techniques, which at first allows for layers of such 2D materials on polymeric surfaces to be isolated by means of mechanical transfer techniques [15]. More on the theoretical side, our setup follows those reported in the literature; density functional theory based methodologies are used to predict interfacial electric field within (mono)layers in van der Waals heterostructures to evaluate (among the others) the photocatalytic performance enhancement [16,17], and the storage of alkali atoms (batteries) [18], while similar approaches are exploited to compute properties of metal/oxide interfaces in terms of workfunction variation and charge distribution between heterostructure components [19].

So, a first step will be to verify the confinement and electrical response of GO layers deposited on a polymeric surface when in contact with water droplets and then validate the models and results once the GO is integrated within a polymeric fiber to also study its mechanical strength for their exploitation in fabrics.

In this study, we embedded GO flakes obtained from liquid suspension into poly(methyl methacrylate) (PMMA)/GO fibers by the coagulation method. Beyond the superior electrical and mechanical properties with respect to the neat polymer, we observed the segregation on the fiber surface of the GO sheets. It was found that water droplet interaction with the GO surface generates an electrostatic potential under ambient conditions. On the theoretical side, we confirmed the electrical charge redistribution associated with the approach of water molecules on a GO layer. We pave the way so that the combinations of our findings could be used to create an electrostatic nanocomposite fabric as protection against the transmission of aerosol particles.

## 2. Materials and Methods

### 2.1. Material Preparation

GO was purchased from Cheaptubes, while poly(methyl methacrylate) (PMMA, average Mw 15.000) was purchased from Sigma Aldrich. The preparation method was reported by us elsewhere [14]; briefly GO water solution (1 mg/mL) was deposited by spin coating (500 rpm) on cleaned quartz substrates and left to dry under nitrogen stream. The PMMA for the realizations of the films was produced through the direct polymerization of the methyl methacrylate (MMA) monomer. It was stirred together with lauroyl peroxide (Sigma Aldrich, [CH_3_(CH_2_)_10_CO]_2_O_2_, 2 wt%) and N,Ndimethyl-m-toluidine (Sigma Aldrich, CH_3_C_6_H_4_N(CH_3_)_2_, 2 wt%), for 10 min. The reacting mixture was poured in a petri dish and positioned in a glass vial with an excess of MMA. PMMA was then spin cast (1000 rpm for 1 min) on GO and annealed for 2 h from 30 °C to 50 °C with a 10 °C/h heating ramp in order to remove residual monomer in the samples; then it was immersed at 100 °C in boiling water until the complete detachment of the PMMA film was obtained. Metal electrodes (≈60 nm) were deposited by vacuum evaporation onto the detached PMMA/GO film. To prepare the PMMA/GO composite fiber, 2 g of PMMA powder was dissolved in 6 g of dimethylformamide (DMF, Sigma Aldrich) and 4 g of tetrahydrofuran (THF, Sigma Aldrich). GO was added at 1.0 wt% into 12 g of the PMMA solution. After stirring the solution for 1 h, it was extruded through a stainless-steel syringe into a coagulation bath with deionized (DI) water at a speed of 50 μL/min.

Commercial beer yeast extract was added to water (50 mg/mL) and stirred in a sterilized flask at 110 rpm at 30 °C for 1 h. After that, sugar three times the weight of the medium was added. Fermenting yeast was then dropped onto the PMMA/GO film that was positioned into a circular aluminum mold.

### 2.2. Material Characterizations

After the PMMA/GO film realization, the current–voltage characterization was performed with a Keithley 2420 source meter; conductivity values were obtained by applying a linear voltage and monitoring the current across the film. A conductive silver paint was deposited on Au thermal evaporated electrodes at the end of rectangular shaped samples with probes that were placed 2 cm apart. The open-circuit voltage (Voc) of the PMMA/GO film was measured after the exposure with a DI water aerosol from a distance between 10 and 30 cm at ambient conditions. The aerosol water was left to evaporate in a calm environment or under air flow with a speed of 1 m/s. In the case of the PMMA/GO fibers, the current–voltage characterization was performed by applying a linear voltage along the fiber and monitoring the current.

Raman spectra were acquired with LabRAM Aramis (HORIBA Jobin Yvon, Lille, France), where the excitation wavelength used was 632.8 nm generated from a He–Ne laser with an output power of approximately 15 mW. The diffraction grating used had 1200 grooves cm^−1^ and the CCD detector Peltier-cooled was at ~−68 °C. The acquisition range for each spectrum was 800 to 3200 cm^−1^ and each spectrum was computed by averaging two successive integrations of 10 s each. The objective lens of the microscope (Olympus BX41) used was a 100× with a long working distance. Aperture slit and pinhole were set respectively to 1000 μm and 100 μm. Kelvin probe force microscopy (KPFM) measurements were carried out on AFM instrument from Bruker (Model: ScanAsyst) in ambient conditions at 33% relative humidity.

The mechanical properties of the PMMA/GO fibers were measured by a universal tensile testing machine (Lloyd Instr. LR30K) with a 500 N cell. The strain rate was 5 mm/min, and the gauge length was 20.0 ± 0.5 mm. Five samples for each composition were tested. The morphology of the PMMA/GO fibers was investigated by field emission scanning electron microscopy (FESEM).

The optical transparency of the film was monitored by UV–Vis spectroscopy, measuring the absorbance between 400 and 1000 nm wavelengths.

### 2.3. Computational Details

Calculations were all performed by means of the density functional theory as implemented in the Vienna Ab-initio Simulation Package (VASP) code [20,21,22,23]. The Perdew–Burke–Ernzerhof (PBE) electron exchange-correlation functional [24], along with the DFT-D3 dispersion correction, in order to include the van der Waals interactions [25,26], were adopted in the calculations. The projector augmented wave (PAW) potentials [27] have been similarly employed with a cutoff energy of 600 eV for the plane-wave basis set. Optimizations converged when forces were below 0.02 eV/Å.

**Figure 1:**
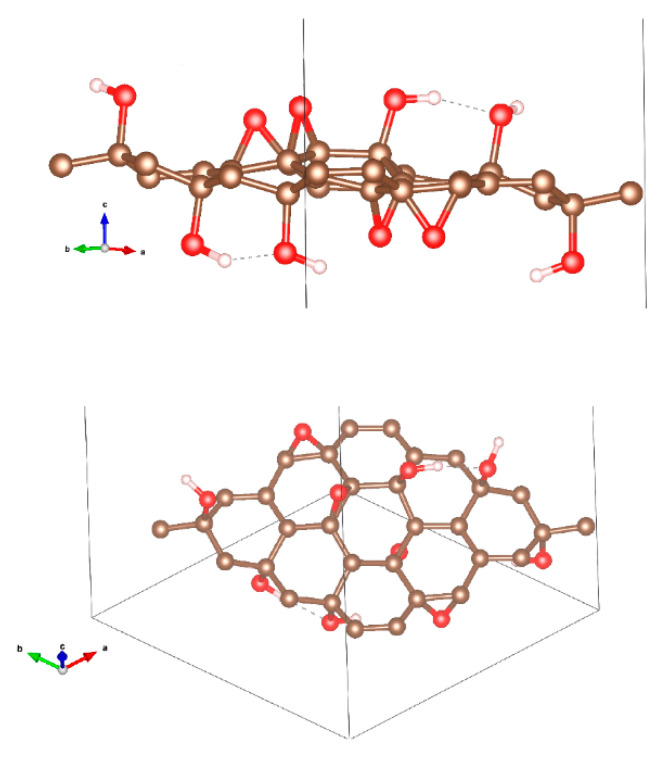
Lateral and top view of optimized structure of the graphene oxide (GO) supercell (brown: C, red: O, and white: H atoms).

The graphene initial atomic structure was taken from the Materials Project website [28]. In detail, the graphite primitive cell was considered and “peeled” to obtain a single-layer. A 4 × 4 supercell was then constructed and optimized (adding a very large amount of vacuum, ~50 Å, along the non-periodic direction to avoid spurious interactions among replicas) and the same number of epoxy and hydroxyl terminations were anchored on the two faces. The choice of the two terminations stems from previous literature that clearly assessed their dominant presence in GO [29,30,31]. The overall ratio OH:epoxy was 3:2. The so-assembled structure was once more fully optimized (both ionic and lattice parameters), obtaining the geometry shown in Figure 1. We are aware that our model is probably limited and may underestimate the real situation, however, similar findings to that in the previous literature makes us confident of the reliability of our results [32]. In particular, to validate our results, we calculated the workfunction (Φ) of GO, which is the difference between the vacuum level (E_vac_) and the Fermi Energy (E_F_): for our analyzed GO system, we obtained a value of 5.43 eV, while that for pure graphene, as a reference, is 4.25 eV, which is in perfect agreement with the value reported by Kumar et al. (4.2 eV) [32].

To simulate water anchoring on the GO layer, we then asymmetrically added, as a test case, a layer of five water molecules on top of the GO layer and once more optimized the so-assembled system. From the initially relaxed structure, we constrained molecules of water to progressively get closer to the GO layer to evaluate the possible dipole enhancement (charge redistribution) at the interface between the two structures. The final charge analysis was performed by means of the Bader code [33,34,35,36].

## 3. Results and Discussion

**Figure 2:**
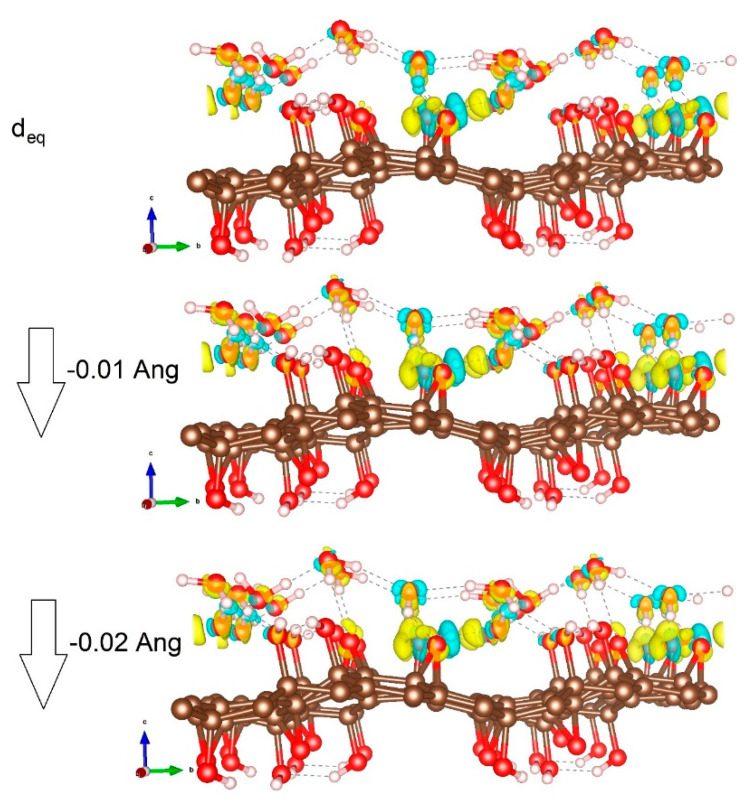
Charge distribution induced by the interface formation. Here, the charge is calculated as the difference between the charge distribution of the coupled structure minus the two contributions, H_2_O and GO layers, respectively, in their interacting geometry. [brown: C, red: O, and white: H atoms. Yellow and light blue isosurfaces are negative and positive charge distribution (isosurface level 0.005 e/Bohr^3^)].

On the theoretical side, from the initial modellization, we evaluated the possible charge redistribution associated with the approach of water molecules on the GO layer. Figure 1 and Figure 2 show the details of the simulation. In details, Figure 2 shows the charge distribution induced by the interface formation. Kelvin probe force microscopy (KPFM) provides information on the contact potential difference between the tip and the sample surface and is associated with local workfunction [37]. The results reported elsewhere [38] indicate a value of nearly 4.7 eV, which is within the value range from 3.7 to 5.1 eV reported for GO and is in good agreement with that calculated by our modellization (see Figure 1) [39].

Inter- and intralayer van der Waals forces dominate the heterostructure as one can clearly see from the figure. The physisorbed water layer is expected in this sense not to transfer charge to the GO layer because of the formal absence of chemical bonds between the two layers. To prove it, we calculated the total charge distribution in the water layer, finding as expected a small variation (−0.056): the O atom charge ranged between −1.08 and −0.97, and that of H between 0.47 and 0.60 [36]. We then focused on the effects of water impact on the GO layer and the associated possible charge distribution variation. Accordingly, we reduced the distance of water oxygen by 0.01 and 0.02 Å (closer distances did not lead to any stable heterostructure), keeping the water O positions frozen and allowing all the other ionic positions (and lattice lateral parameters) to relax. The result was an overall change in the net charge of the water molecule layer that in both cases turned to be slightly more negative at −0.075 and −0.083, respectively. Of course, this is not a very sensitive variation, but is still noticeable, and moreover, we could observe a trend indicating an enhanced dipolar redistribution due to the pressure exerted by water on the GO layer, which is supportive of the change in the electric field associated with the interface formation.

**Figure 3:**
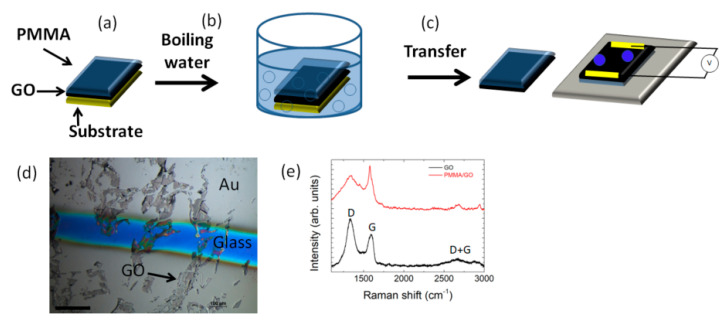
Scheme of transfer method of (GO). (**a**) poly(methyl methacrylate) (PMMA) was deposited on GO. (**b**) The substrate was then immersed in boiling water to promote the detachment of PMMA. (**c**) Transfer onto the target substrate. (**d**) Optical image of PMMA/GO on the target glass substrate between gold electrodes; the scale bar indicates 100 µm. (**e**) Raman spectra of GO and PMMA/GO film from 1000 cm^−1^ to 3200 cm^−1^ showing the characteristics D, G, and D + G peaks.

The steps for the transfer method using low molecular weight PMMA as the support layer is illustrated in Figure 3. After coating GO with the PMMA solution (Figure 3a), the PMMA/GO film was peeled off in boiling water (Figure 3b), which was used to weaken the adhesion between the PMMA and substrate. The floating PMMA/GO film was transferred onto the target substrate, heated at 105 °C glass transition to improve the adhesion with the substrate, and then subjected to electrical characterization (Figure 3c). Figure 3d shows GO flakes of micron size on a glass substrate that can be identified by their clear contrast with respect to the substrate.

With respect to our previous findings [14,15], the employment of a PMMA support layer with a lower molecular weight reduced the contamination of GO; in this regard, the Raman spectrum does not show any peak associated with PMMA, being the peaks of D (~1335 cm^−1^) and G (~1588 cm^−1^), the signature of the sp3 and sp2 hybridized carbon state, respectively (Figure 3e) [40]. The D peak appears due to the disorder and imperfection of the carbon crystallites and the G peak is assigned to one of the two E2g modes during the stretching vibration of the graphitic carbon atoms [40]. The D + G bump is activated by the presence of defects in the GO paper [41].

**Figure 4:**
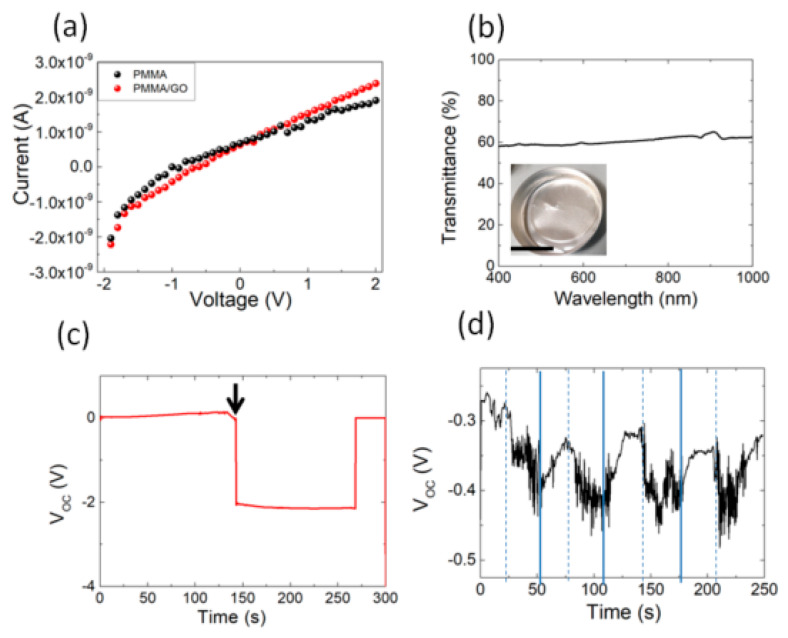
(**a**) Current-voltage characteristic plots of PMMA and PMMA/GO films. (**b**) UV–Vis transmittance spectrum of the PMMA/GO film. The inset shows the photograph of the PMMA/GO film into an aluminum mold (the scale bar indicates 2 cm). (**c**) Open-circuit voltage (V_oc_) change during water droplet evaporation (the arrow indicates the instant of time in which the drops of water come into contact with the sample) from PMMA/GO films under ambient conditions. (**d**) V_OC_ change under constant air speed (≈1 m/s) in the laboratory environment. The dashed lines indicate when the air flow was turned on. The continuous lines indicate when the aerosol was switched on.

The absorption of water molecules on nanostructured carbon layers along with fluid evaporation is known to play a crucial role in the generation of electrical potentials [42,43]. After peeling, the PMMA/GO film is electrically insulating (Figure 4a) and transparent (Figure 4b); after being in contact with water droplets, it shows an open-circuit voltage (Voc) change up to −2 V and then recovers the initial baseline after an interval between 150 and 250 s (Figure 4c). By repeating the experiment in dynamic conditions (i.e., by wetting and drying the film with air flow (Figure 4d)), we observed a change of the open circuit voltage under the water evaporation with a variation that was smaller than that observed in Figure 4c.

**Figure 5:**
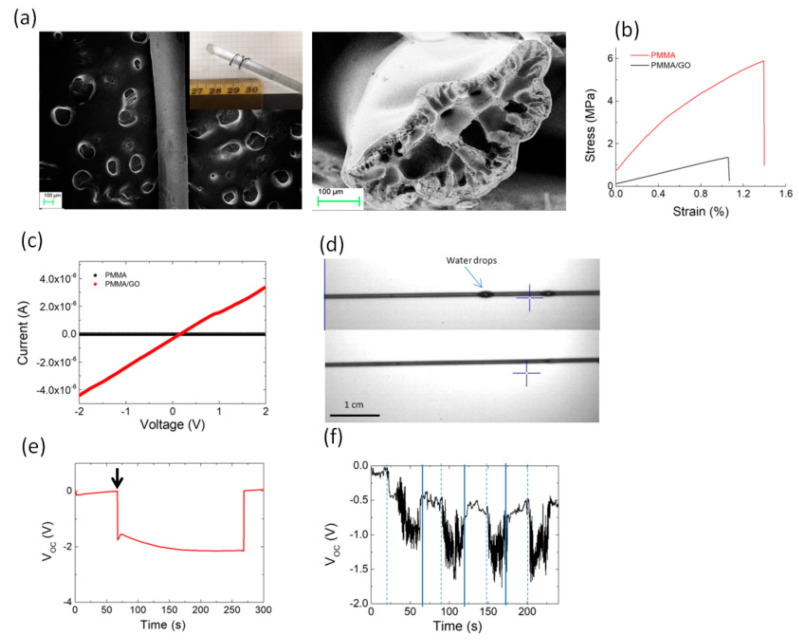
(**a**) Field emission scanning electron microscopy (FESEM) image of PMMA/GO fiber showing the surface and the cross section of the PMMA/GO fiber. The inset shows the photograph of the fabricated PMMA/GO composite microfiber wound on a glass rod. (**b**) Representative stress–strain curves for PMMA and PMMA/GO fibers produced by the coagulation method. (**c**) Electrical conductivity for PMMA and PMMA/GO fibers, respectively. (**d**) Optical visualization of water droplets on PMMA/GO fiber (upper panel) and after 30 s of evaporation (lower panel). (**e**) Open-circuit voltage (V_oc_) measured during water aerosol evaporation from the PMMA/GO fiber under ambient conditions (the arrow indicates the instant of time in which the drops of water come into contact with the sample). (**f**) V_OC_ change under constant air speed (≈1m/s) in the laboratory environment. The dashed lines indicate when the air flow was turned on. The continuous lines indicate when the aerosol was switched on.

Figure 5 shows the morphology, mechanical, and electrical properties of PMMA/GO fibers obtained by coagulation. The fabricated PMMA/GO fiber had a diameter of about 200 μm (inset of Figure 5a,b). The FESEM image revealed that the surface was fairly smooth with the presence of GO aggregates (Figure 5a). In general, the FESEM image of the cross section showed hollow pores in the radial direction of the fiber. The formation of this structure is attributed to counter diffusion of solvent/nonsolvent and phase separation during coagulation [44]. From a mechanical point of view, the addition of GO resulted in an improvement of the strength as well as of the elongation at break, leading to a toughness (calculated as the area under the stress-strain curve) variation from 0.13 MPa for PMMA to 0.34 MPa for the PMMA/GO fibers, respectively (Figure 5b). Contrary to what has been observed for the PMMA/GO film (Figure 4a), the addition of GO in the fiber generates a considerable increase in electrical conductivity (Figure 5c). The highest electrical conductivity and the improvement of the mechanical properties of fibers when GO was added can be explained by an increase in polymer chain orientation and GO orientation along the fiber direction, increasing the electrical current flow along the fiber.

The water adsorption in hygroscopic materials was found a green method of converting the evaporation into electric potential [45]. The water confinement on the PMMA/GO fiber is shown in Figure 5d. Graphene oxide flakes with abundant functional groups on the fiber surface make it hydrophilic, so that water capture occurs along the fiber. After being wetted by an aerosol with deionized water, an open-circuit voltage (Voc) of −2 V was recorded, maintaining this value during the water evaporation in the laboratory environment with the temperature of 22 °C (Figure 5e). We then repeated the experiment by wetting and drying the fiber with air flow (Figure 5f). When the fiber was exposed to water aerosol, a variation of the voltage was observed up to −1.5 V. It returned to its original value when we turned on the air flow, promoting the water evaporation. Such wetting- and drying-induced voltage change has been observed on several cycles (Figure 5f).

This finding mimics the electrostatic interactions that were observed in natural and synthetic fabrics [46,47]. Recently, for instance, a charged polyvinylidene fluoride (PVDF) multilayer nanofiber was exploited in filtering airborne coronavirus under ambient nano-aerosols [48].

In the absence of novel coronavirus (COVID19) to test, we investigated the fermentation process of the beer yeast (*S. Cerevisiae*) on the PMMA/GO film. We chose beer yeast cells because their surface is negatively charged [49] and has the spike proteins of coronaviruses [50].

**Figure 6:**
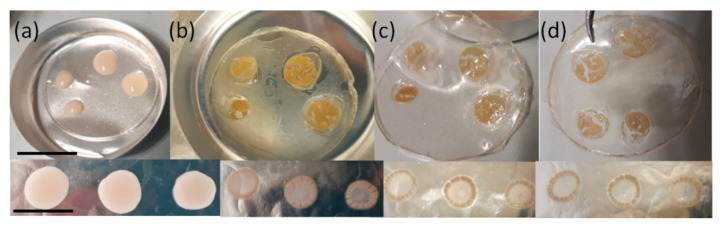
(**Top**) Yeast cell biofilm formation on PMMA/GO film at different times: (**a**) as deposited, (**b**) 10 s, (**c**) 50 s, and (**d**) 100 s. The scale bar indicates 2 cm. (**Bottom**) Yeast cell biofilm formation on PMMA film at different times: (**a**) as deposited, (**b**) 10 s, (**c**) 50 s, and (**d**) 100 s. The scale bar indicates 0.5 cm.

The effectiveness of the PMMA/GO film against yeast cells is shown in Figure 6. Qualitative and macroscopic observation of yeast cells on the PMMA/GO substrate revealed that under the water evaporation, namely 10 s, 50 s, and 100 s, led to the presence of transparent regions, indicating fewer cells stained. Similar observations on the neat PMMA substrate used as the control did not give evidence of transparent regions, suggesting a good adhesion of the biofilm on the substrate (Figure 6, bottom). This qualitative investigation of biofilm formation could suggest the efficacy of the composite film against airborne microorganisms.

## 4. Conclusions

We exploited a well-known method used to transfer graphene layers to create a composite surface that could generate a negative voltage from water evaporation. First-principles calculations support the idea that water molecules, when approaching a graphene oxide layer, enhance a dipolar redistribution at the interface, thus generating an extra electric field due to the heterostructure formation. Experiments on the wet surface revealed a drying-induced open circuit voltage change. A similar effect was observed on PMMA/GO fibers that were fabricated by the wet spinning method. Considering the electrostatic interaction with the spike proteins of coronaviruses, these materials could be considered for integration in personal protection equipment against airborne viruses transported by aerosols.

## References

[1] Yin J., Li X., Yu J., Zhang Z., Zhou J., Guo W. (2014). Generating electricity by moving a droplet of ionic liquid along grapheme. Nat. Nanotechnol..

[2] Xue G., Xu Y., Ding T., Li J., Yin J., Fei W., Cao Y., Yu J., Yuan L., Gong L. (2017). Water-evaporation-induced electricity with nanostructured carbon materials. Nat. Nanotechnol..

[3] Liu G., Chen T., Xu J., Wang K. (2018). Blue energy harvesting on nanostructured carbon materials. J. Mater. Chem. A.

[4] Zhao F., Cheng H., Zhang Z., Jiang L., Qu L. (2015). Direct power generation from a graphene oxide film under moisture. Adv. Mater..

[5] Dreyer D.R., Park S., Bielawski C.W., Ruoff R.S. (2010). The chemistry of graphene oxide. Chem. Soc. Rev..

[6] Zhu Y., Murali S., Cai W., Li X., Won Suk J., Potts J.R., Ruoff R.S. (2010). Graphene and graphene oxide: Synthesis, properties, and applications. Adv. Mater..

[7] Joshi R.K., Carbone P., Wang F.C., Kravets V.G., Su Y., Grigorieva I.V., Wu H.A., Geim A.K., Nair R.R. (2014). Precise and ultrafast molecular sieving through graphene oxide membranes. Science.

[8] Wei N., Peng X., Xu Z. (2014). Understanding water permeation in graphene oxide membranes. ACS Appl. Mater. Interfaces.

[9] Mouhat F., Coudert F.-X., Bocquet M.-L. (2020). Structure and chemistry of graphene oxide in liquid water from first principles. Nat. Commun..

[10] Ye S., Shao K., Li Z., Guo N., Zuo Y., Li Q., Lu Z., Chen L., He Q., Han H. (2015). Antiviral Activity of Graphene Oxide: How Sharp Edged Structure and Charge Matter. ACS Appl. Mater. Interfaces.

[11] Konda A., Prakash A., Moss G.A., Schmoldt M., Grant G.D., Guha S. (2020). Aerosol Filtration Efficiency of Common Fabrics Used in Respiratory Cloth Masks. ACS Nano.

[12] Shen B., Zhai W.T., Tao M.M., Lu D.D., Zheng W.G. (2013). Chemical functionalization of graphene oxide toward the tailoring of the interface in polymer composites. Compos. Sci. Technol..

[13] Nguyen L., Choi S.M., Kim D.H., Kong N.K., Jung P.J., Park S.Y. (2014). Preparation and characterization of nylon 6 compounds using the nylon 6-grafted GO. Macromol. Res..

[14] Valentini L., Bittolo Bon S., Kenny J.M. (2013). Liquid Droplet Excitation of Freestanding Polymethylmethacrylate/Graphene Oxide Films for Mechanical Energy Harvesting. J. Polym. Sci. Part B Polym. Phys..

[15] Valentini L., Bittolo Bon S., Kenny J.M. (2013). Polymethyl methacrylate/Graphene Oxide Layered Films as Generators for Mechanical Energy Harvesting. ACS Appl. Mater. Interfaces.

[16] Masuda Y., Giorgi G., Yamashita K. (2014). DFT study of anatase—Derived TiO_2_ nanosheets/graphene hybrid materials. Phys. Status Solidi B.

[17] Liu J. (2015). Origin of High Photocatalytic Efficiency in Monolayer g-C_3_N_4_/CdS Heterostructure: A Hybrid DFT Study. J. Phys. Chem. C.

[18] Zheng Y., Zhou T., Zhao X., Pang W.K., Gao H., Li S., Zhou Z., Liu H., Guo Z. (2017). Atomic Interface Engineering and Electric—Field Effect in Ultrathin Bi_2_MoO_6_ Nanosheets for Superior Lithium Ion Storage. Adv. Mater..

[19] Prada S., Martinez U., Pacchioni G. (2008). Work function changes induced by deposition of ultrathin dielectric films on metals:A theoretical analysis. Phys. Rev. B.

[20] Kresse G., Hafner J. (1993). Ab initio molecular dynamics for open–shell transition metals. Phys. Rev. B.

[21] Kresse G., Hafner J. (1994). Ab initio molecular–dynamics simulation of the liquid–metal–amorphous–semiconductor transition in germanium. Phys. Rev. B.

[22] Kresse G., Furthümller J. (1996). Efficiency of ab–initio total energy calculations for metals and semiconductors using a plane–wave basis set. Comput. Mater. Sci..

[23] Kresse G., Furthmüller J. (1996). Efficient iterative schemes for ab initio total–energy calculations using a plane–wave basis set. Phys. Rev. B.

[24] Perdew J.P., Burke K., Ernzerhof M. (1996). Generalized gradient approximation made simple. Phys. Rev. Lett..

[25] Grimme S., Antony J., Ehrlich S., Krieg H. (2010). A consistent and accurate ab initio parametrization of density functional dispersion correction DFT-D for the 94 elements H-Pu. J. Chem. Phys..

[26] Grimme S., Ehrlich S., Goerigk L. (2011). Effect of the damping function in dispersion corrected density functional theory. J. Comput. Chem..

[27] Blöchl P. (1994). Projector augmented-wave method. Phys. Rev. B.

[28] Materials Project. https://materialsproject.org/materials/mp-568286/#.

[29] Loh K.P., Bao Q., Eda G., Chhowalla M. (2010). Graphene Oxide as a Chemically Tunable Platform for Optical Applications. Nat. Chem..

[30] Bagri A., Mattevi C., Acik M., Chabal Y.J., Chhowalla M., Shenoy V.B. (2010). Structural Evolution during the Reduction of Chemically Derived Graphene Oxide. Nat. Chem..

[31] Gao W., Alemany L.B., Ci L., Ajayan P.M. (2009). New Insights into the Structure and Reduction of Graphite Oxide. Nat. Chem..

[32] Kumar P.V., Bernardi M., Grossman J.C. (2013). The Impact of Functionalization on the Stability, Work Function, and Photoluminescence of Reduced Graphene Oxide. ACS Nano.

[33] Tang W., Sanville E., Henkelman G. (2009). A grid-based Bader analysis algorithm without lattice bias. J. Phys. Condens. Matter.

[34] Sanville E., Kenny S.D., Smith R., Henkelman G. (2007). An improved grid-based algorithm for Bader charge allocation. J. Comp. Chem..

[35] Henkelman G., Arnaldsson A., Jónsson H. (2006). A fast and robust algorithm for Bader decomposition of charge density. Comput. Mater. Sci..

[36] Yu M., Trinkle D.R. (2011). Accurate and efficient algorithm for Bader charge integration. J. Chem. Phys..

[37] Palermo V., Palma M., Samorì P. (2006). Electronic Characterization of Organic Thin Films by Kelvin Probe Force Microscopy. Adv. Mater..

[38] Tripathi M., Valentini L., Rong Y., Bittolo Bon S., Pantano M.F., Speranza G., Guarino R., Novel D., Iacob E., Liu W. Free-standing hybrid paper of graphene oxide and functionalized carbon nanotube for enhanced electrical and mechanical properties.

[39] Ji S., Min B.K., Kim S.K., Myung S., Kang M., Shin H.-S., Song W., Heo J., Lim J., An K.-S. (2017). Work function engineering of graphene oxide via covalent functionalization for organic field-effect transistors. Appl. Surf. Sci..

[40] Malard L.M., Pimenta M.A., Dresselhaus G., Dresselhaus M.S. (2009). Raman spectroscopy in graphene. Phys. Rep..

[41] Cançado L.G., Jorio A., Ferreira E.H.M., Stavale F., Achete C.A., Capaz R.B., Moutinho M.V.O., Lombardo A., Kulmala T.S., Ferrari A.C. (2011). Quantifying defects in graphene via Raman spectroscopy at different excitation energies. Nano Lett..

[42] Liu Z., Zheng K., Hu L., Liu J., Qiu C., Zhou H., Huang H., Yang H., Li M., Gu C. (2010). Surface-Energy Generator of Single-Walled Carbon Nanotubes and Usage in a Self-Powered System. Adv. Mater..

[43] Ding T., Liu K., Li J., Xue G., Chen Q., Huang L., Hu B., Zhou J. (2017). All-Printed Porous Carbon Film for Electricity Generation from Evaporation-Driven Water Flow. Adv. Funct. Mater..

[44] Qin O.Y., Chen Y.S., Zhang N., Mo G.M., Li D.H., Yan Q. (2011). Effect of jet swell and jet stretch on the structure of wet-spun polyacrylonitrile fiber. J. Macromol. Sci. Part B Phys..

[45] Chen X., Goodnight D., Gao Z., Cavusoglu A.H., Sabharwal N., DeLay M., Driks A., Sahin O. (2015). Scaling up nanoscale water-driven energy conversion into evaporation-driven engines and generators. Nat. Commun..

[46] Perumalraj R. (2015). Characterization of Electrostatic Discharge Properties of Woven Fabrics. J. Text. Sci. Eng..

[47] Frederick E.R. (1986). Fibers, Filtration and Electrostatics—A Review of the New Technology. J. Air Pollut. Control Assoc..

[48] Leung W.W.-F., Sun Q. (2020). Charged PVDF multilayer nanofiber filter in filtering simulated airborne novel coronavirus (COVID-19) using ambient nano-aerosols. Sep. Purif. Technol..

[49] Leather R.V., Dale C.J., Morson B.T. (1997). Characterisation of beer particle charges and the role of particle charge in beer processing. J. Instr. Brew..

[50] Yao Q., Masters P.S., Ye R. (2013). Negatively charged residues in the endodomain are critical for specific assembly of spike protein into murine coronavirus. Virology.

